# Epilepsy: knowledge, attitude and awareness in Jeddah Saudi Arabia

**DOI:** 10.1186/1471-2164-15-S2-P61

**Published:** 2014-04-02

**Authors:** Deena Fuad Haneef, Heyam Abdulsamad Abdulqayoum, Ahad Abdullah Sherbeni, Muhammad Faheem, Adeel G Chaudhary, Mohammad H Al-Qahtani, Muhammad Imran Naseer

**Affiliations:** 1 Department of Medical Laboratory, Faculty of Applied Medical Sciences, King Abdulaziz University, Jeddah, Saudi Arabia; 2 Department of Biochemistry, Faculty of Science, King Abdulaziz University, Jeddah, Saudi Arabia; 3 Center of Excellence in Genomic Medicine Research (CEGMR), King Abdulaziz University, Jeddah, 21589, Saudi Arabia

## Background

Epilepsy is a neurological disorder affecting individuals of all ages and very common in Saudi Arabia with a prevalence of 6.54 per 1000 [1]. This study was conducted to evaluate general knowledge, public awareness and attitude towards epileptic patients in Jeddah, Saudi Arabia.

## Materials and methods

A survey composed of 22 questions concerning the neurological disorder was used to collect data from 1122 samples of random residents of Jeddah, Saudi Arabia. Distribution methods included a hand-to-hand method and the creation of an online version of the survey which was published through the web.

## Results

Total of the 1122 respondents who completed the survey, 1023 (91%) reported their awareness of the disorder, 514 (46%) witnessed an epileptic seizure during their lifetime and 715 (64%) stated their unawareness of handling a patients with an epileptic seizure. A total of 985 (88%) reported that epilepsy is not an infectious disease, 712 (63%) believed that epilepsy is not related to evil or Jin’s possession, 794 (71%) accepted sharing of schools with epileptic patients, 877 (78%) believed that an epileptic patient is capable of living a normal life, although 548 (49%) refused to marry an epileptic patient. Additionally, 1067 (95%) showed their encouragement to build and support the existence of an organization that will support epileptic patients.

## Conclusions

Our results showed that epilepsy is a well-known disorder in Saudi Arabia but the level of awareness was very low and it can be improved by educating the people. Residents of Jeddah seemed to accept epileptic patients for their condition but still clearly unaware of what the neurological disorder truly means. This survey showed that most of the responses were due to misunderstanding of the disorder. Hence, basic educational awareness programs and campaigns are needed for building an epileptic friendly society. Furthermore, this survey will also provide a base for international comparison of epilepsy with Saudi Arabia.
